# Assessing connection to outpatient care among patients screening positive for previously undiagnosed diabetes in the emergency department: a combined retrospective cohort and prospective cohort survey study

**DOI:** 10.1186/s12913-026-14935-y

**Published:** 2026-06-10

**Authors:** Christian A. Koziatek, Vidhyasai Annem, Aaron Chan, Joline Fong, Harita Reddy, Reed Caldwell, Robert Femia, Zoe Grabinski, David C. Lee

**Affiliations:** 1 Ronald O. Perelman Department of Emergency Medicine, NYU Grossman School of Medicine, New York City, NY USA; 2 Department of Emergency Medicine, Bellevue Hospital Center, New York City, NY USA; 3 College of Natural Sciences, University of Texas at Austin, Austin, TX USA; 4 Department of Pediatrics, NYU Grossman School of Medicine, New York City, NY USA; 5 Department of Population Health, NYU Grossman School of Medicine, 227 East 30th Street, Room 107, New York City, NY 10016 USA

**Keywords:** Diabetes, Screening, Emergency department, Follow-up, Access to care

## Abstract

**Background:**

Patients with frequent emergency department (ED) use often have poor connection to primary care, limiting access to preventive screening. Screening patients at risk for diabetes is essential to ensure early treatment via medication and lifestyle changes. While ED screening programs have shown success in diagnosing diabetes, it is unknown the impact on follow-up with subsequent care, and thus downstream patient outcomes. This study aimed to evaluate the efficacy of an emergency department (ED)-based diabetes screening program, assessing follow-up for subsequent management, if patients changed health behaviors or initiated medications, and barriers reported to follow-up if patients did not establish care.

**Methods:**

Screening results and patient data from four ED sites of a health system were retrospectively collected; we then performed a phone-based survey of all patients diagnosed with diabetes via ED screening over one year. Patients were queried about diagnosis awareness, connection to follow-up or barriers to establishing care, and health behavior changes (diet, exercise, medications).

**Results:**

Of 9,897 screened ED patients, 615 (6.2%) had diabetes. We surveyed 307 patients (50% response rate). Among newly diagnosed patients; 79% reported obtaining outpatient care, but only 46% recalled ED diagnosis. Health behavior changes included 65% reporting diet improvement, 35% increasing exercise, and 52% starting diabetes medication. Patients who failed to follow-up reported barriers including awareness of screening result, time/scheduling issues, and financial considerations.

**Conclusion:**

This study assesses the reported rate of outpatient care following diabetes diagnosis via an ED screening program and identifies barriers experienced by patients who did not obtain follow-up. Improved connections between screening programs and subsequent care are needed to improve outcomes.

**Supplementary Information:**

The online version contains supplementary material available at 10.1186/s12913-026-14935-y.

## Background

Diabetes is a leading cause of morbidity and mortality in the United States. Early detection is crucial for proper treatment and management, but nearly 1 in 4 adults with diabetes are undiagnosed [1, 2]. Further, risk factors for diabetes overlap with risk factors for lack of regular access to primary care primary care [3]. Patients who lack primary care access often utilize emergency departments (EDs) to manage their healthcare needs, with nearly 20–30% of Americans reporting the ED as their primary source of healthcare [4–6]. These patients are more likely to be younger, male, Black or Hispanic, uninsured or underinsured, or lack housing [7–10]. The ED therefore offers a unique opportunity to engage high-risk patients by offering preventive screening that might normally be delivered in primary care settings [11].

In 2019, our health system implemented an ED-based diabetes screening initiative testing hemoglobin A1c (HbA1c) for patients without a documented history of diabetes in accordance with US Preventative Service Task Force guidelines [12]. Previous evaluations of this program and other reported ED-based screening programs have demonstrated success in identifying previously undiagnosed diabetes amongst screened ED patients [12–15]. However, lack of follow-up has been a challenge in translating this success to downstream patient outcomes [11, 13, 16–18].

This study aimed to evaluate the efficacy of the ED-based diabetes screening program, assessing whether newly diagnosed patients were able to follow-up for care with a primary care provider, and if patients changed health behaviors or initiated treatment – and if not, what obstacles they reported to doing so. Assessing the effectiveness of non-primary care-based diabetes screening initiatives is important not only to improve the diagnosis of diabetes in high-risk populations, but to connect high-risk patients to downstream outpatient care and ultimately improve patient outcomes.

## Methods

### Study design, setting, and population

We performed a study of all ED patients at the New York University Langone Health system who were screened for diabetes between January 1st, 2022 and December 31st, 2022 and found to have a HbA1c of 6.5% or higher. A combined methodology of retrospective cohort analysis and prospective patient surveys was employed. The screening program was based in four EDs (an academic, university tertiary care center, a community level 1 trauma center hospital, a free-standing community ED, and an academic community hospital ED) in New York City with a total annual volume of approximately 400,000 patient encounters per year.

ED patients were selected for a screening HbA1c if they were already receiving bloodwork in the ED as part of their evaluation, aged 35 to 70 years old, with body-mass-index (BMI) of 25 or higher, without any electronic health record (EHR) previous diagnosis of diabetes (based on problem list within our health system or auto-populated via the shared health record system Care Everywhere [Care Everywhere LLC, Natck, MA] which queries participating outside electronic health records for patients, or by patient-provided past medical history at ED triage), and with no HbA1c result in the EHR in the last six months based on both inpatient and outpatient data. For patients whose screening led to a HbA1c result consistent with diabetes, they received direct counseling from their ED provider team regarding their new diagnosis and/or a call from our ED follow-up center by a nurse or advanced practice provider to make them aware of the laboratory results and the need to connect with a primary care provider for further care. Additional details and results of the screening program have been previously reported [12]. EHR data was queried from the Epic Systems Clarity database (Epic Systems Corporation, Verona, Washington) with the use of Oracle SQL Developer (Oracle Corporation, Redwood City, California) and exported for data analysis; the queried data included encounter ID, patient age, sex, race/ethnicity, payor (e.g., insurance) type, primary language, and screening HbA1c result.

### Follow-up survey

To perform follow-up surveys among the ED patients screened in 2022 who had a HbA1c of 6.5% or higher, a group of 9 volunteer research assistants contacted patients by phone at least 6 months after their HbA1c testing in the ED. For patients whose primary language was not English, interpreter services (Pacific Interpreters, Monterrey, CA) were used. Each patient received up to 3 phone calls (unless patients hung up or requested no further contact). A scripted voicemail was left for patients who were not reached but had an answering service. Response rates were tracked.

The survey was modelled on the Agency for Healthcare Research and Quality Consumer Assessment of Healthcare Providers and Systems (CAHPS) ED Survey for follow-up care, with modifications made by context experts in emergency medicine, social work, and population health. It was divided into four sections with questions related to: (1) the diagnosis of diabetes, (2) ability to follow-up for outpatient care, (3) management of diabetes for those who followed-up and barriers to care among those who did not follow-up, and (4) changes in their health and behaviors since the diagnosis (Supplemental Fig. 1) [19].

Regarding the diagnosis of diabetes, patients were asked if they remember being diagnosed and if it was a new diagnosis. For follow-up outpatient care, patients were asked if they were able to follow-up, and if so, in what time frame and setting (primary care, endocrinology, or other practice). For patients who followed-up, we asked whether they were started on any medications, follow-up HbA1c testing, and regularity of healthcare visits since screening diagnosis. Patients reporting no follow-up were asked about the main reason they did not follow-up, and whether any of the following factors were also a problem: awareness, knowledge, insurance, cost, time, availability, scheduling, or language. Research assistants also recorded responses to open-ended questions regarding barriers to obtaining outpatient follow-up care. All patients, regardless of follow-up status, were asked whether they had made changes to lifestyle or health behaviors including diet, exercise, or diabetes medication use.

### Study outcomes

The primary study outcome was whether ED patients with newly diagnosed diabetes successfully reported follow-up with outpatient care. While ED patients were selected for diabetes screening as previously described, some patients who were screened reported a previously known diagnosis of diabetes; those patients were excluded from the analysis. Secondary outcomes included determining patient awareness of diagnosis, follow-up HbA1c testing amongst those with outpatient follow-up, medication initiation for diabetes management, changes in health behaviors (i.e., diet or exercise) since screening, time to follow-up, and specialist care. For those who did not establish outpatient follow-up, barriers to follow-up were queried and recorded.

### Statistical analysis

Descriptive statistics were used to analyze patient demographic information and screening results. For survey data, we analyzed differences in response rates and comparisons for follow-up or behavior changes amongst groups using t-tests for continuous variables and the Pearson chi-squared test for categorical variables. Groups for comparison included geographic area stratified by location of ED, and severity of disease stratified by index screening HbA1c result – 9.0% or higher vs. 6.5–8.9%. Statistical significance was set at a *p*-value of 0.05. Descriptive statistics and data analysis were performed using Stata V14.2 (StataCorp, College Station, TX). Survey response rate was calculated using the AAPOR (American Association for Public Opinion Research) Response Rate 2 (RR2) calculation [20].

### Ethics and consent to participate

This study was approved by the Institutional Review Board at the New York University School of Medicine. A waiver of authorization and consent was issued for the study and the consent requirement was waived by the IRB. This study was also carried out in compliance with the Helsinki Declaration regarding human subjects research.

## Results

A total of 9,897 ED patients were screened during the study period; 615 (6.2%) had a HbA1c of 6.5% or higher consistent with diabetes. The study team successfully contacted 307 patients, for an AAPOR response rate of 50% (RR2 calculation). Common reasons for unsuccessful contact included inability to reach patient by phone or patients declining to participate. We did not identify any statistically significant differences between responders and non-responders in terms of age (*p* = 0.27), sex (*p* = 0.24), race/ethnicity (*p* = 0.17), language (*p* = 0.49), or health insurance (*p* = 0.20). Table 1 reports survey response rates and participant characteristics. There was no significant difference in response rates across ED sites (*p* = 0.31). Differences in race and ethnicity groups amongst respondents reflected known demographics of the ED location, with a higher number of Black respondents seen in the Manhattan site, and a higher number of Hispanic-Latino respondents seen at the Brooklyn site. There were no significant differences in glycemic control or payor type between the ED sites. Amongst survey respondents, 41% recalled their ED screening results as consistent with diabetes; 23% recalled being tested but did not recall results, and 33% did not recall testing.

**Table 1:** Survey response rates and participant characteristics by screening ed site

Screening and Follow-up Care	All Sites	Manhattan	Brooklyn	Long Island	Significance
**Diagnosed in 2022**	615	117	305	193	
**All Survey Participants**	307	51	157	99	
**Survey Response Rate**	50%	44%	51%	51%	0.31
**Respondent Demographics**					
Average Age	54	56	52	56	< 0.01
**Sex**					
Male	344 (56%)	64 (55%)	168 (55%)	112 (58%)	0.78
Female	271 (44%)	53 (45%)	137 (45%)	81 (42%)	
**Race/Ethnicity**					
White	96 (31%)	21 (41%)	33 (21%)	42 (42%)	< 0.01
Black	76 (25%)	20 (39%)	34 (22%)	22 (22%)	
Hispanic	93 (30%)	6 (12%)	67 (43%)	20 (20%)	
Asian	20 (7%)	3 (6%)	7 (4%)	10 (10%)	
Other	22 (7%)	1 (2%)	16 (10%)	5 (5%)	
**Primary Language**					
English	237 (77%)	47 (92%)	106 (67%)	84 (85%)	< 0.01
Spanish	60 (20%)	2 (4%)	45 (29%)	13 (13%)	
Other	10 (3%)	2 (4%)	6 (4%)	2 (2%)	
**Health Insurance**					
Private	65 (21%)	14 (27%)	30 (19%)	21 (21%)	0.36
Medicare	25 (8%)	4 (8%)	10 (6%)	11 (11%)	
Medicaid	30 (10%)	4 (8%)	18 (12%)	8 (8%)	
Managed Care	172 (56%)	26 (51%)	88 (56%)	58 (58%)	
Self-pay	15 (5%)	3 (6%)	11 (7%)	1 (1%)	
**Respondent HbA1c Results**					
HbA1c 6.5% to 8.9%	197 (64%)	33 (65%)	93 (59%)	71 (72%)	0.13
HbA1c 9.0% or higher	110 (36%)	18 (35%)	64 (41%)	28 (28%)	
**Respondent Recall of Result**					
Diabetes	125 (41%)	26 (51%)	67 (43%)	32 (32%)	0.07
Prediabetes	10 (3%)	1 (2%)	2 (1%)	7 (7%)	
Normal	1 (0%)	0 (0%)	0 (0%)	1 (1%)	
Remembered testing but not result	70 (23%)	11 (22%)	32 (20%)	27 (27%)	
Did not remember being tested	101 (33%)	13 (25%)	56 (36%)	32 (32%)	
**Prior History of Diabetes**					
Yes, Not in EHR Records	132 (44%)	21 (42%)	77 (50%)	34 (35%)	0.06
No, Newly Diagnosed	171 (56%)	29 (58%)	77 (50%)	64 (65%)	

*P* values are included for each category

While inclusion criteria for the ED-based screening program included absence of a documented history of diabetes in the EHR, 44% (132) of patients with a HbA1c of 6.5% or above reported they had a known history of diabetes that was not reflected in the EHR. Therefore, 171 patients (56%) confirmed they had no prior diagnosis (Table 1). Successful follow-up was reported by 79% of all survey respondents with newly diagnosed diabetes. There was no significant difference in outpatient follow-up rates, dietary, or exercise health behavior changes between those patients who screened in with HbA1c 6.5–8.9% versus those with HbA1C ≥ 9.0% at the time of ED screening (Table 2).

**Table 2:** Survey responses among ED patients with newly diagnosed diabetes by level of glycemic control

	All Newly Diagnosed Cases	HbA1c 6.5% to 8.9%	HbA1c >= 9.0%	Significance
Number of Survey Respondents	171 (100%)	104 (61%)	67 (39%)	
**Remembered HbA1c Test Result**	78 (46%)	44 (42%)	34 (51%)	0.28
Outpatient Follow-Up Obtained	133 (79%)	78 (76%)	55 (82%)	0.38
Improved Dietary Behaviors	111 (65%)	66 (63%)	45 (67%)	0.62
Improved Exercise Behaviors	59 (35%)	38 (37%)	21 (31%)	0.49

*P-*values are included for each category

### Outcomes among newly diagnosed patients who had outpatient follow-up care

Among patients who reported follow-up with outpatient care after diagnosis in the ED, 59% had HbA1c of 6.5% to 8.9%, versus 41% with HbA1C ≥ 9.0%. Patients with HbA1c ≥ 9.0% reported significantly faster follow-up intervals after diagnosis (*p* = 0.02) and were more likely to follow-up with endocrinology either alone or in addition to primary care (*p =* 0.01). A majority of patients were started on some form of medication; patients with HbA1c ≥ 9.0% were more frequently started on insulin either alone or in combination with oral medications (*p* < 0.01). However, 20% of newly diagnosed patients reported not being started on any diabetes medications at all (Table 3).

**Table 3:** Survey responses among ED patients with newly diagnosed diabetes who had outpatient follow-up care

	All Newly Diagnosed Cases	HbA1c 6.5% to 8.9%	HbA1c >= 9.0%	Significance
**Number of Survey Respondents**	133 (100%)	78 (59%)	55 (41%)	
**Timing of Outpatient Follow-Up**				
Within 1 Week	40 (31%)	21 (28%)	19 (36%)	0.02
Within 1 Month	46 (36%)	21 (28%)	25 (47%)	
Within 3 Months	30 (23%)	25 (33%)	5 (9%)	
Within 6 Months	5 (4%)	4 (5%)	1 (2%)	
Within 12 Months	8 (6%)	5 (6%)	3 (6%)	
**Outpatient Follow-Up Type**				
Primary Care	85 (66%)	57 (76%)	28 (53%)	0.01
Endocrinology	20 (16%)	8 (10%)	12 (23%)	
Primary Care & Endocrinology	17 (13%)	5 (7%)	12 (23%)	
Other Care Setting	6 (5%)	5 (7%)	1 (2%)	
**Initiation of Medications**				
Started on Oral Medications	68 (52%)	46 (60%)	22 (40%)	< 0.01
Started on Insulin Injections	16 (12%)	4 (5%)	12 (22%)	
Started on Oral and Insulin	21 (16%)	5 (7%)	16 (29%)	
No Medications Started	26 (20%)	21 (28%)	5 (9%)	
**Frequency of Follow-Up**				
1 Visit Since Diagnosis	10 (8%)	6 (8%)	4 (8%)	0.04
2 Visits Since Diagnosis	28 (22%)	20 (26%)	8 (15%)	
3 Visits Since Diagnosis	33 (25%)	24 (31%)	9 (17%)	
4 + Visits Since Diagnosis	58 (45%)	27 (35%)	31 (60%)	
**Follow-Up HbA1c Testing**				
Yes, with improvement	84 (64%)	46 (60%)	38 (70%)	0.24
Yes, but no change	11 (9%)	9 (12%)	2 (4%)	
Yes, but worse control	5 (4%)	4 (5%)	1 (2%)	
Yes, but result unknown	8 (6%)	4 (5%)	4 (7%)	
No, definitely no repeat	16 (12%)	8 (10%)	8 (15%)	
Unknown, not sure if tested	7 (5%)	6 (8%)	1 (2%)	
**Change in Health Behaviors**				
Improved Dietary Behaviors	94 (71%)	56 (72%)	38 (70%)	0.86
Improved Exercise Behaviors	53 (40%)	35 (45%)	18 (33%)	0.18

*P-*values are included for each category. Not all totals sum to the total number of survey respondents; a small number of respondents did not answer each question

Most patients reported multiple outpatient follow-up visits after diagnosis, with nearly half reporting 4 or more; patients with HbA1c ≥ 9.0% were more likely to have 4 or more visits (*p* = 0.04) compared to those with HbA1c of 6.5% to 8.9%. The majority of patients reported having repeat HbA1c testing; 64% reported improvement in HbA1c, with no difference seen by degree of glycemic control (*p* = 0.24) (Table 3).

### Outcomes among newly diagnosed patients who did not follow-up for outpatient care

Table 4 reports survey results among the 36 patients who reported no follow-up outpatient care after ED screening diagnosis. The most commonly reported barriers to outpatient care were time (31%), awareness (25%), scheduling (25%), insurance (22%), and appointment availability (17%). When stratified by level of glycemic control, patients with HbA1c ≥ 9.0% were significantly less likely to report a lack of awareness of diagnosis (*p* = 0.01). Patients who did not follow-up for outpatient care reported fewer changes in health behaviors compared to those who did follow-up for both diet and exercise (47% and 17% respectively) (*p* < 0.01) (Table 4).

**Table 4:** Survey responses among ED patients with newly diagnosed diabetes who were unable to follow-up for outpatient care

	All Newly Diagnosed Cases	HbA1c 6.5% to 8.9%	HbA1c >= 9.0%	Significance
**Number of Survey Respondents**	36 (100%)	24 (67%)	12 (33%)	
**Follow-Up Barriers Reported**				
Awareness	9 (25%)	9 (38%)	0 (0%)	0.01
Knowledge	3 (8%)	3 (13%)	0 (0%)	0.21
Insurance	8 (22%)	5 (21%)	3 (25%)	0.78
Cost	5 (14%)	4 (17%)	1 (8%)	0.50
Time	11 (31%)	6 (25%)	5 (42%)	0.31
Availability	6 (17%)	5 (21%)	1 (8%)	0.34
Scheduling	9 (25%)	7 (29%)	2 (17%)	0.41
Language	2 (6%)	1 (4%)	1 (8%)	0.61
**Changes in Health Behaviors**				
Improved Dietary Behaviors	17 (47%)	10 (42%)	7 (58%)	0.35
Improved Exercise Behaviors	6 (17%)	3 (13%)	3 (25%)	0.34

*P-*values are included for each category

## Discussion

This study of ED-based screening for diabetes explores the ability of newly diagnosed patients to establish appropriate downstream care. In our sample of survey respondents, 79% of newly diagnosed patients established subsequent outpatient care. These results provide insights to patient outcomes following a new diagnosis of diabetes made in the ED, including dietary and exercise behavior changes amongst those who did follow-up and positive trends in reported HbA1c levels for the majority of these. For those who did not follow-up after diagnosis in the ED, a spectrum of barriers including awareness of the diagnosis, time to find care, and cost issues/access to health insurance were reported.

Prior studies of ED-based diabetes screening have examined follow-up rates, although without exploring next steps in treatment care or outcomes. Prior to the establishment of HbA1c criteria for the diagnosis of diabetes, ED screening programs attempted to establish high-suspicion populations amongst ED patients via either elevated random serum glucose values or HbA1c values, and then encouraged patients to return for an oral glucose tolerance test. In a study in Boston, 38% of patients followed-up for the confirmatory testing [11]; in a Los Angeles study, 48% did so [13]. Subsequent studies using HbA1c criteria drawn in the ED, confirming a new diagnosis in real time for ED patients, reported a follow-up rate of 55% [18] or did not report on follow-up rates.

Our results show a higher percentage of surveyed patients obtained follow-up, though differences in surveyed patients in likelihood to seek outpatient care may account for differences from prior research. Notably, a separate study estimating the impact of ED-based screening asked potential patients if they planned follow-up for further testing and treatment, for which 95% reported intent to seek care. Subsequent findings of substantially lower rates of follow-up at the same site highlight both recall issues and barriers to follow up as we identified in this initiative [21]. Failure to connect to outpatient care after diagnosis has been identified as a primary barrier in other diabetes community screening programs as well, outside the ED [22]. Our results also emphasize the importance of establishing outpatient care for newly diagnosed diabetes patients; they were more likely to improve their diets, exercise more, and the majority reported an improved HbA1c.

In addition to awareness of the HbA1c result, commonly reported barriers to establishing outpatient care included time constraints, difficulty scheduling appointments, insurance issues, and availability of appointments. For instance, one study participant stated that “life just got in the way”, and “since I didn’t feel sick, I didn’t feel the need to go see a doctor”. Several newly diagnosed patients reported attempting to make appointments but did not receive a response or next available appointments were months away. Several study participants reported concerns about cost and lack of health insurance; one reported that they were not able to get diabetes medications, as they had no primary doctor. Lack of time was also a frequently reported barrier, due to work, caring for other family members, or other time demands. Prior work examining barriers to diabetes care after an ED visit in patients with known diabetes who were found to by hyperglycemic similarly found that patients reported financial considerations, lack of understanding or communication issues during the ED visit/discharge process, and trouble obtaining outpatient care [23]. These barriers require attention to improve follow-up rates and treatment in order to achieve improved downstream patient-centered outcomes; potentially utilizing dedicated discharge counseling if screening positive, social work interventions in the ED, financial counseling, specified follow-up pathways for screening program patients, and other interventions known to improve connection to outpatient care from the ED [24–26]. Notably, awareness of diagnosis was a barrier for follow-up for those who screened in with a moderately elevated HbA1c, but not for patients with significantly elevated HbA1c; it is possible a more abnormal lab result may have prompted a more memorable or thorough counseling in the ED by care teams at the time of diagnosis.

Patients with poor access to primary care are known to have higher rates of all-cause mortality, increased prevalence of chronic disease, and higher rates of hospitalization and complication from chronic disease [27–31]. ED-based screening programs therefore may be an important aspect in improving community health, especially amongst vulnerable/high-risk populations, by decreasing delays in diagnosis of chronic disease where earlier treatment and lifestyle change are crucial for health outcomes, like diabetes [32, 33]. However, bridging the gap from ED visit to follow-up care is critical to successfully initiating downstream treatment and improving patient outcomes. While counseling in the ED or from a follow-up call after visit care center is probably beneficial, nearly half the patients in our study did not recall any screening test at all or did not recall a positive screening result. Furthermore, among both those who did obtain follow-up care and those who did not, a combined lack of awareness or knowledge of screening result was highly prevalent. An emphasis on counseling and discharge planning is therefore an area of emphasis for future efforts, although efficiency trade-offs and time and environmental constraints on ED providers and staff are well documented and present an obstacle [34, 35]. Successfully addressing this problem may require dedication of additional resources or leveraging new technologies like telemedicine [36, 37].

Our study has a number of limitations. First, as is a known limitation of any survey collected data, the inability to establish contact with and survey about half of patients who screened positive in the ED limits the generalizability of the findings due to a potential response bias [38]. It is possible patients who answered the survey were also more likely to seek follow-up care, follow medical recommendations, or otherwise have different behaviors from those who were not reached. Second, it is possible that patients inaccurately reported on their subsequent HbA1c values, or their medications or health behaviors. Additionally, while we had a strong survey response rate, ultimately a relatively few number of newly diagnosed patients who did not obtain follow-up care answered questions regarding barriers to care and health behavior changes (*n* = 36). In our survey to study participants, we did not assess for certain negative consequences of failure to connect to primary care, such as recurrent ED visits or hospitalizations. It will be important in future studies to note these outcomes as connection to primary care might very well reduce the need for frequent emergency department visits and prevent hospitalizations. Finally, it is worth noting the limitation of EHR data for the implementation of screening; a very high number of patients screened on the basis of having no past medical history of diabetes in the health record reported a known history of diabetes, limiting the specificity and yield of the ED-based screening program.

## Conclusions

This study reports the frequency that patients report establishing outpatient care after a new diagnosis of diabetes was made in the ED and examines key gaps reported by patients in connecting to downstream diabetes care, including awareness of the screening results and financial or scheduling barriers reported by patients who did not follow-up. These results can inform efforts to improve the linkage of ED-based diabetes screening programs to outpatient longitudinal care.

## Supplementary Information

Below is the link to the electronic supplementary material.

Supplementary Material 1

## Data Availability

The datasets used and analyzed during the current study are available from the corresponding author on reasonable request.
