# Supplementary material for: Assessing connection to outpatient care among patients screening positive for previously undiagnosed diabetes in the emergency department: a combined retrospective cohort and prospective cohort survey study
Source: BMC Health Serv Res. 2026 Jun 10;26:1134. doi: 10.1186/s12913-026-14935-y (PMC13480042; doi:10.1186/s12913-026-14935-y)
Supplement: Supplementary file 1 [file 12913_2026_14935_MOESM1_ESM.pdf]

Screening Form

[redacted] Medical Record Number
(Double Check Entry for Errors) ______________________

Patient Name ______________________

Preferred Language ______________________

Home Phone ______________________

Emergency Department ______________________

Date of Emergency Visit ______________________

Health Insurance ______________________

Screening HbA1c Result ______________________

Result Category
- ◯ Diabetes
- ◯ Prediabetes
- ◯ Normal

Research Associate

Day and Time Called ______________________

Does the patient remember having a test for diabetes in the ED last year, and if so, then do they remember what their result meant?
- ◯ Diabetes
- ◯ Prediabetes
- ◯ Normal
- ◯ Remembers test but not result
- ◯ Does not remember being tested
- ◯ Patient hung up or refused to respond
- ◯ Phone number invalid or incorrect

Was this a new diagnosis of diabetes / prediabetes or was it already known by the patient or their primary care provider (or endocrinologist)?
- ◯ Yes
- ◯ No

Was the patient able to follow up with a primary care provider (or endocrinologist) about their diabetes test result?	◯ Yes<br>◯ No
Approximately when was the patient able to get to the primary care provider (or endocrinologist) for follow up?	◯ Within 1 week<br>◯ Within 1 month<br>◯ Within 3 months<br>◯ Within 6 months<br>◯ Within 12 months<br>◯ After 12 months
Where did you follow for care after being screened for diabetes?	☐ [redacted] primary care<br>☐ [redacted] endocrinologist<br>☐ [redacted] other practice<br>☐ non-[redacted] primary care<br>☐ non-[redacted] endocrinologist<br>☐ non-[redacted] other practice
If so, then what did their primary care provider (or endocrinologist) suggest that they do about their test result?	________________________________
Were they started on any medications for diabetes by their primary care provider (or endocrinologist)?	◯ Yes - Only Oral Medications<br>◯ Yes - Oral Medications and Insulin<br>◯ Yes - Only Insulin Medications<br>◯ No - No Medications for Diabetes
Have they had a follow-up HbA1c test since being screened for diabetes in the ED?	◯ Yes - HbA1c result better<br>◯ Yes - HbA1c result unchanged<br>◯ Yes - HbA1c result worse<br>◯ Yes - HbA1c result unknown<br>◯ No - Definitely not retested<br>◯ Unknown - Not sure if retested
How regularly have the patients visit been with their primary care provider (or endocrinologist) since being screened?	◯ One visit since being screened<br>◯ Two visits since being screened<br>◯ Three visits since being screened<br>◯ Four or more visits since being screened
If not, what was the main reason that they had trouble following up with a primary care provider (or endocrinologist)?	________________________________
Were any of these factors also a problem in trying to find a primary care provider (or endocrinologist) for follow up?	☐ Awareness - Not aware of an abnormal HbA1c result<br>☐ Knowledge - Not sure what the abnormal result meant<br>☐ Insurance - Difficulty due to type of healthcare insurance<br>☐ Cost - Cost of healthcare visits or medications prescribed<br>☐ Time - No time required to go to a healthcare appointment<br>☐ Availability - Trouble making an visit that is soon enough<br>☐ Scheduling - Not sure about how they would make a visit<br>☐ Language - Barriers due to language in scheduling a visit

Were there any other challenges that made it hard to see a primary care provider (or endocrinologist)?	____
Was there anything that our healthcare team at [redacted] could have done to help them get to an appointment?	____
What changes in their health (if any) did they make after they received their HbA1c result?	☐ Diet - Changed their diet because of the HbA1c test<br>☐ Exercise - Changed their exercise because of the HbA1c<br>☐ Medication - Started a medication because of the HbA1c
Did they make any other changes in their health behaviors since being tested for diabetes?	____
Did you find it helpful to be screened for diabetes during your emergency department visit?	____
